# Sensing the load

**DOI:** 10.7554/eLife.50210

**Published:** 2019-10-07

**Authors:** Nele Haelterman, Joohyun Lim

**Affiliations:** Department of Molecular and Human GeneticsBaylor College of MedicineHoustonUnited States

**Keywords:** Piezo1, mechanosensitive ion channel, mechanotransduction, bone formation, osteoblast, osteocyte, Human, Mouse

## Abstract

How does the skeleton detect and adapt to changes in the mechanical load it has to carry?

**Related research article** Sun W, Chi S, Li Y, Ling S, Tan Y, Xu Y, Jiang F, Li J, Liu C, Zhong G, Cao D, Jin X, Zhao D, Gao X, Liu Z, Xiao B, Li Y. 2019. The mechanosensitive Piezo1 channel is required for bone formation. *eLife* **8**:e47454. doi: 10.7554/eLife.47454

**Related research article** Li X, Han L, Nookaew I, Mannen E, Silva MJ, Almeida M, Xiong J. 2019. Stimulation of Piezo1 by mechanical signals promotes bone anabolism. *eLife* **8**:e49631. doi: 10.7554/eLife.49631

The adult skeleton is an extremely dynamic tissue that can adjust its mass and strength to exactly the values needed for efficient movement. For instance, the bones in the dominant arm of a tennis player have a greater mineral density than the bones in their non-dominant arm (Huddleston, 1980). However, bone matter can also be lost if muscles do not get used enough (or if they do not have to support the body against gravity, as happens in space). The ability of bone to sense the mechanical load it has to support, and to adapt to changes in its environment, are central to the body's ability to build the exact amount of bone needed to prevent it from breaking as a result of increased muscle tension or external trauma. But how does bone sense the mechanical load that it carries?

Although the details remain unclear, cells called osteocytes are known to have a prominent role in sensing loads. Osteocytes are descendants of osteoblasts – the cells that build new bone. Whereas osteoblasts reside on the bone surface to deposit new bone, osteocytes are buried deep within mature bone (Figure 1; Long, 2012). Here, they regulate the activity of both osteoblasts and cells called osteoclasts that are responsible for bone resorption (the process by which old bone is broken down). Previous studies have shown that osteocytes alter their transcriptional profile in response to changes in biomechanical strain. For instance, an increase in load is associated with a reduction in the expression of a gene called *Sost*, which is known to prevent bone formation by osteoblasts (Robling et al., 2008). However, despite much progress, we still do not fully understand how bone is able to sense changes in mechanical load.

**Figure 1.**
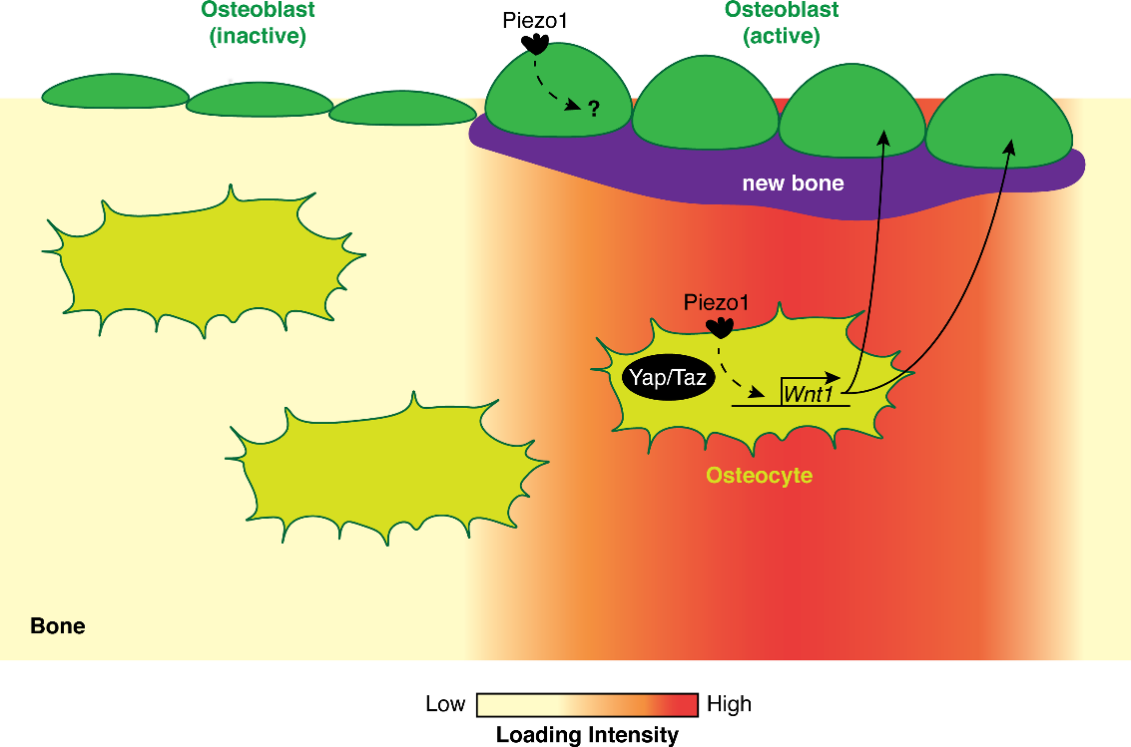
How biomechanical loading stimulates bone formation. In regions of bone that experience high biomechanical loads (red), the Piezo1 ion channel is activated in both osteoblasts (green) and osteocytes (olive). In osteocytes, the activation of Piezo1 leads to increased Wnt1 expression in a Yap/Taz-dependent manner. Wnt1 then activates the Wnt signaling pathway, which stimulates the formation of new bone (purple) by osteoblasts. In regions of bone that experience average or low biomechanical loads, the osteoblasts are inactive and there is no increase in bone mass.

Now, in eLife, two independent groups report that an ion channel called Piezo1 has a central role in the process of mechanosensation (Li et al., 2019; Sun et al., 2019). Piezo1 is a protein embedded in the cell membrane that responds to mechanical stimulation by changing the influx of calcium and other positive ions. In the first paper, Bailong Xiao (Tsinghua University), Yingxian Li (Chinese Astronaut Research and Training Centre) and co-workers – including Weijia Sun and Shaopeng Chi as joint first authors – report the results of a series of experiments on Piezo1 (Li et al., 2019). First, they observed that the precursors of osteoblasts only respond to mechanical stimulation (which in this case involved 'poking' the cell membrane with a glass pipette) when Piezo1 is present. They also showed that the Piezo1 channel is essential for these cells to differentiate into functional osteoblasts. Next, Sun et al. explored the role played by Piezo1 in vivo by genetically engineering mice in which a signal could be used to delete Piezo1 in osteoblasts and osteocytes. These animals had normal bone resorption, but showed impaired bone strength, formation and structure.

Are these effects due to Piezo1 being a mechanosensitive ion channel? To answer this question, Sun et al. first showed that osteoblasts which lack Piezo1 no longer show the mechanically-induced ion currents seen in osteoblasts that are derived from wild-type mice. More importantly, when the skeleton of animals lacking Piezo1 expression in osteoblasts and osteocytes is exposed to either an increase or a reduction in mechanical load, the associated responses in bone mass are blunted. This shows that the Piezo1 channel is necessary to sense the biomechanical load and to change the rate at which bone is formed.

In the second paper Jinhu Xiong (University of Arkansas for Medical Sciences) and co-workers – including Xuehua Li as first author – also report new findings on Piezo1 (Sun et al., 2019). They placed osteocytes under fluid shear stress (which is a different kind of mechanical stimulation) and used RNA-seq to examine changes in gene expression for a range of calcium channels: the largest changes were observed in Piezo1. Mice in which this channel had been deleted from osteoblasts and osteocytes also showed reduced bone mass and biomechanical strength, confirming the role of Piezo1 in osteoblasts and their descendants. (It is worth noting that the two groups used different approaches to delete Piezo1, but still came to similar conclusions.) Increasing the load on the bones of these mutant animals no longer led to increased bone mass or osteoblast activity, indicating that the channel is at least partially required to 'translate' the signal of increased biomechanical load into increased bone mass.

How would activation of Piezo1 lead to the formation of new bone? Li et al. found that deleting Piezo1 reduces the expression of a signaling protein called Wnt1, which is critical for bone formation (Joeng et al., 2017). In addition, their results suggest that Piezo1 enhances the expression of Wnt1 by increasing the activity of Yap and Taz, two mechanosensitive transcription cofactors that are required for bone formation (Figure 1; Kegelman et al., 2018; Xiong et al., 2018). Lastly, when a drug called Yoda1 was used to activate Piezo1 in adult wild-type mice, the long bones of these animals showed an increase in both Wnt1 levels and bone mass.

Together, the results of Sun et al. and Li et al. strongly suggest that Piezo1 plays a key role in helping the skeleton respond to changes in mechanical loading. However, the results also raise intriguing questions. First, it remains unclear whether Piezo1 mainly mediates mechanosensation in osteoblasts or in osteocytes because we do not have the genetic tools needed to answer these questions. Second, we still do not know if there is a link between the mechanosensing ability of the Piezo1 channel and its role in the differentiation of osteoblasts and osteoprogenitor cells involved in the formation and maintenance of bone (Sugimoto et al., 2017). Third, Piezo1 may not be the only mechanosensitive channel that plays a role in mediating the response of bone to changes in mechanical stimulation. Fourth, we do not fully understand the Yap/Taz-Wnt axis: while Li et al. show that Piezo1 stimulates Wnt1 expression in part through Yap/Taz, it is unclear if this is a direct consequence of Piezo1 activation.

Finally, showing that the effects of biomechanical loads on bone can be mimicked by pharmaceutically activating Piezo1 could be a promising step towards the development of treatments for bone conditions such as osteoporosis. However, we need to proceed carefully as increased Piezo1 expression can stimulate cell death in cartilage and lead to osteoarthritis (Lee et al., 2014), underscoring the need for a complete understanding of the mechanosensation pathway in bone and cartilage.
